# Network pharmacology-based and experimental identification of the effects of Renshen Yangrong decoction on myocardial infarction

**DOI:** 10.3389/fphar.2022.1010036

**Published:** 2022-10-25

**Authors:** Jiahao Zhao, Xing Xu, Xiaolong Yang

**Affiliations:** ^1^ Regenerative Medicine Research Center, West China Hospital, Sichuan University, Chengdu, China; ^2^ Core Facilities of West China Hospital, Sichuan University, Chengdu, China; ^3^ West China School of Basic Medical Sciences and Forensic Medicine, Sichuan University, Chengdu, China

**Keywords:** myocardial infarction, Renshen Yangrong decoction, reactive oxide species, TNF-α, IL-6, network pharmacology

## Abstract

**Objective:** Myocardial infarction (MI) is one of the leading causes of death worldwide. Currently, the drugs used to treat MI have various side effects. Emerging evidence supports the protective effects of Renshen Yangrong Decoction (RSYRD) in cardiovascular diseases (CVDs) treatments, with few side effect reports. However, the role of RSYRD in MI remains unclear. In this study, network pharmacological analysis was combined with experiments *in vivo* and *in vitro* to validate the effects of RSYRD in the treatment during the early stage of MI.

**Methods:** Firstly, network pharmacology analysis was performed to search for the potential targets and signaling pathways of RSYRD in the early stage of MI. Then, the protein-protein interaction (PPI) network was constructed to identify the core genes of RSYRD that may play a key role in MI. At last, the treatment effectiveness of RSYRD on MI was verified *via* experiments *in vitro* and *in vivo*.

**Results:** RSYRD contained fifty-six bioactive components. Eighty-eight intersections between RSYRD and MI targets and thirteen core genes were screened. KEGG and GO functional enrichment analyses predicted that RSYRD might play a therapeutic role in MI through oxidative stress, apoptosis, and immune-inflammatory signaling pathways. *In vivo* and *in vitro* experiment results revealed that significant apoptosis occurred in myocardial tissue in the early stage of MI. Moreover, the levels of reactive oxide species (ROS), TNF-α, and IL-6 increased markedly. After RSYRD administration, they significantly decreased. At the mechanistic level, RSYRD could reduce ROS production to alleviate cell apoptosis.

**Conclusion:** RSYRD could reduce neonatal mouse cardiomyocytes (NMCMs) apoptosis by lowering ROS production induced by hypoxia and improve the cardiac function of mice 3 days post-MI. RSYRD could also reduce the levels of TNF-α and IL-6 in the serum of mice.

## 1 Introduction

Cardiovascular diseases (CVDs) are the leading causes of death worldwide (Mensah et al., 2019; Pickersgill et al., 2022). In 2019, they caused 18.6 million deaths and doubled the disability rate among survivors of cardiovascular disease (Roth et al., 2018). The costs of CVDs treatment impose a substantial economic burden on society. Myocardial infarction (MI) is one of the significant ischemic heart diseases (Gelosa et al., 2022). When the coronary artery is blocked or stenosed, the blood supply to the heart is reduced or stopped, eventually causing MI (Vogel et al., 2019; Cohen et al., 2019). Following MI, the ultrastructure of heart cells is changed, and long-term hypoxia causes cell death. Apoptosis, one type of cell death, plays a crucial role in the early stage of MI (Nicotera and Melino, 2004; Konstantinidis et al., 2012).

Drugs that can inhibit platelet aggregation, statins that can reduce blood lipids, and anticoagulant drugs that can prevent thrombosis are currently used clinically to treat MI. However, recent clinical trials have demonstrated that the effects of antithrombin drugs on reducing arterial blockage are inconclusive. Statins have had toxic effects on liver function, and current thrombolytic drugs in achieving immediate or long-term vascular patency during treatment have limited effects. (Gershlick and More, 1998; Thompson et al., 2016). In contrast, numerous research studies have found that traditional Chinese medicine (TCM) has distinct advantages in the treatment of CVDs (Hao et al., 2017; Yang et al., 2019; Liu et al., 2021; Chen et al., 2022; Jiang et al., 2022; Testai et al., 2020).

Furthermore, numerous studies have reported the treatment effectiveness of TCM on MI, such as Shen Yuan Dan attenuates hypoxia-induced cardiomyocyte injury, reduces lactate dehydrogenase activities, increases cell viability, and reduces cardiomyocyte apoptosis in neonatal rats (Chu et al., 2022); Danqi reduces tachyarrhythmias, enhances left ventricular systolic and diastolic functioning, and decreases border zone fibrosis in post-MI rats (Ma et al., 2019). Based on the growing evidence, TCM appears to be one of the most important treatments for MI. Renshen Yangrong Decoction (RSYRD), composed of Radix Paeoniae Alba, Radix Angelicae Sinensis, Pericarpium Citri Reticulate, Tragacanth, Radix Ginseng, Rhizoma Atractylodis Macrocephalae, and Poria cocos, is primarily used to promote hematopoietic function and immune function. Moreover, it is also utilized to treat coronary heart disease, diabetic complications, malignant tumors, and brain injuries. Previous research have indicated that RSYRD can inhibit platelet activity in patients with ischemic heart disease and promote the proliferation, migration, and DNA synthesis of human umbilical vein endothelial cells (Sheng et al., 2019). Remarkably, RSYRD has been shown to promote angiogenesis and cerebral protection following ischemic brain injury by activating the HIF/VEGF/NOTCH signaling pathway *via* miRNA-210 (Liang et al., 2021). These findings suggest that RSYRD may have a protective role in MI. To investigate whether RSYRD has a therapeutic effect on MI and its potential mechanism, network pharmacology and *in vivo* and *in vitro* experiments were performed.

## 2 Materials and methods

### 2.1 Bioactive ingredients in Renshen Yangrong decoction

In clinical treatment, drugs are usually taken orally. Oral bioavailability (OB) and drug similarity (DL) are the main factors affecting drug absorption in the gastrointestinal mucosa. Therefore, we used “Radix Paeoniae Alba”, “Radix Angelicae Sinensis”, “Pericarpium Citri Reticulate”, “Tragacanth”, “Radix Ginseng”, “Rhizoma Atractylodis Macrocephalae” and “Poria cocos” as keywords to screen the active ingredients and targets of RSYRD in the Systematic Pharmacology of Traditional Chinese Medicine (TCMSP) (https://www.tcmsp-e.com/) (Ru et al., 2014), with the criteria of OB > 30% and DL > 0.18 (Feng et al., 2018).

### 2.2 Targets related to bioactive ingredients and myocardial infarction

Targets associated with MI were collected from databases including GeneCards (https://www.genecards.org/) (Stelzer et al., 2016), OMIM (https://omim.org/) (Amberger and Hamosh, 2017), PharmGkb (https:// www.pharmgkb.org/), TTD (http://db.idrblab.net/ttd/) (Li et al., 2018), and DrugBank (https://www.drugbank.com/) (Wishart et al., 2006). Using “myocardial infarction” as the keyword, we obtained 4685, 33, 112, 40, and 142 targets from these databases. Then, these targets were normalized in Uniprot (https://www.uniprot.org/).

### 2.3 Network construction

We drew a Venn diagram using an R script summarizing the intersections between RSYRD and MI. A protein-protein interaction (PPI) network of intersections was created using the online STRING database (https://string-db.org/) (Szklarczyk et al., 2019). The CytoNCA plugin of Cytoscape (Version 3.9.1) (https://cytoscape.org/download.html/) was used to identify the hub genes in the intersections we obtained above (Shannon et al., 2003). GO (http://www.geneontology.org/) and KEGG (https://www.genome.jp/kegg/) functional enrichment analyses of intersections, presented in bar graphs and bubble graphs, were performed using the ClusterProfiler package of the R platform (Rstudio, United States, Version 4.1.2) (https://www.rstudio.com/products/rstudio/), with *p* < 0.05 screening criteria (Yu et al., 2012).

### 2.4 Laboratory animals

The specific pathogen-free adult C57BL/6 mice (22–25 g) were purchased from Chengdu Dashuo Experimental Animal Co., LTD (SCXK (Sichuan) 2020-030, China). These mice were fed for 1 week at room temperature (20°C–25 °C) and humidity (54 ± 3%).

### 2.5 Inducing myocardial infarction in a mouse model

After 1 week of feeding, the mice were allocated into MI and sham-operation (SHAM) groups. In the MI group, mice’s left anterior descending coronary artery (LAD) was ligated with 7-0 sutures after 1.5% isoflurane anesthesia, whereas in the SHAM group, only thoracotomy was performed. Three days after surgery, a small animal ultrasound was used to detect the heart function. Afterward, the hearts were made into paraffin and frozen sections for H&E, Masson, and ROS fluorescent staining. Western blotting (WB) was used to detect the apoptosis proteins extracted from the infarct site of mice hearts in the MI group and the corresponding sites in the SHAM group.

### 2.6 Laboratory mice grouping

RSYRD, purchased from the Chengdu University of Traditional Chinese Medicine (Chengdu, China), was soaked in 1000 ml ultrapure water for 60 min, condensed and refluxed for 80 min, and concentrated to 200 ml before being diluted with ultrapure water to a final concentration of 2 g/ml. To investigate the effects of RSYRD on MI, experimental mice were divided into four groups: the SHAM group, in which the mice with thoracotomy were intragastrically administered normal saline (NS); the SHAM + RSYRD group (SHAM + R), in which the mice with thoracotomy were intragastrically administered with RSYRD; the LAD ligated group (MI), in which the mice with LAD ligation were intragastrically administered NS; and the MI + RSYRD group (MI + R), in which the mice with LAD ligation were intragastrically administered with RSYRD. According to previous studies, RSYRD was intragastrically administered at a dose of 38 g/kg per day to the SHAM + R and MI + R groups for 3 days following surgery (Liang et al., 2021).

### 2.7 Detection of laboratory mice’s cardiac function

Mice were washed with phosphate buffer saline (PBS) after hair removal from the chest. The clean mice, anesthetized by 1.5% isoflurane, were fixed on a plate while the instrument monitored heart rate. When the heart rate stabilized at approximately 400, the heart function of the mice was detected by a small animal ultrasonic detector (Vevo platform, probe MX550D (30 MHz), B-mode and M-mode, short axis).

### 2.8 H&E and Masson staining of laboratory mice

The heart tissues were fixed in 10% formalin solution for 48 h after being washed with PBS to remove blood. Following ethanol dehydration, the tissues were immersed in liquid paraffin before slicing. After dewaxing and rehydration, paraffin sections were stained with hematoxylin and eosin for H&E staining and eosin and hematoxylin for Masson staining.

### 2.9 IHC and IF staining of laboratory mice

After antigen retrieval, paraffin sections were immersed in 3% H_2_O_2_ for 20 min to inactivate endogenous peroxidase. The sections were incubated with cleaved caspase 3 (CC3) primary antibody (Cell Signaling Technology, 1:200) overnight at 4°C, followed by blocking with 5% bovine serum albumin (BSA) (Beyotime, China) at room temperature for 45 min. Then, a secondary antibody coupled with horseradish peroxidase (HPR) (Cell Signaling Technology, 1:500) was added and incubated at room temperature for 30 min the next day. DAB kits (Cell Signaling Technology, United States) were used for chromogenic reactions, and hematoxylin was used for nuclear staining. The detailed procedure was performed according to the kit instructions.

The procedures for IF staining were the same as those for IHC. The α-Actinin (Sigma-Aldrich, 1:200) and CC3 primary antibodies were added to the tissues and incubated overnight at 4°C. Then, the secondary fluorescence antibody was used to amplify the signal. Finally, DAPI (Beyotime, China) was added to stain the cell nucleus for 3 min.

### 2.10 Preparation of drug-containing serum

Adult healthy mice were randomly divided into the CONTROL or RSYRD groups. Mice in the RSYRD group were intragastrically given RSYRD at a dose of 38 g/kg per day for 3 days, while mice in the CONTROL group were given NS at the same amount. One hour after the last dose, the mice were anesthetized. Blood was drawn from an abdominal artery and placed at room temperature for 2 h before being centrifuged for 20 min (2000 × *g*). The serum was collected, inactivated at 56°C for 30 min, filtered through a 0.22 μm filter (Millipore, United States), and then stored at −80°C.

### 2.11 Neonatal mouse cardiomyocytes grouping

The method for isolating neonatal mouse cardiomyocytes (NMCMs) from newborn (1–3 day-old) C57BL/6 mice (Dashuo, China) was described in the literature (Makkos et al., 2020; Onódi et al., 2022). NMCMs were cultured in DMEM (High glucose) (BI, Israel) supplemented with 10% fetal bovine serum (FBS) (BI, Israel), 100 U/ml penicillin (Gibco, United States) and 100 g/ml streptomycin (Gibco, United States) as well as humidified air containing 5% CO_2_ at 37°C. After culturing for 48 h, NMCMs were divided into a normoxia control group (Normoxia) and a hypoxia group (Hypoxia) with hypoxia stimulation conditions: glucose-free DMEM (Gibco, United States), 1% O_2_, 94% N_2_, and 5% CO_2_, cultured for 24 h.

To investigate the effects of RSYRD, NMCMs were divided into normoxia control serum group (Normoxia), normoxia drug-containing serum group (Normoxia + R), hypoxia control serum group (Hypoxia), and hypoxia drug-containing serum group (Hypoxia + R). The conditions of hypoxia stimulation were identical to those described in the cell injury model above.

To investigate the effects of RSYRD on ROS, NMCMs were allocated into the hypoxia control serum group (Hypoxia), hypoxia drug-containing serum group (Hypoxia + R), and hypoxia drug-containing serum group + ROS activator (BestBio, BB-47058-1, China) (Hypoxia + R + RD).

### 2.12 Western blotting

Total proteins were quantified by BCA (Beyotime, China) and then transferred to a polyvinylidene fluoride (PVDF) membrane (Millipore, United States) after being separated *via* 12.5% SDS polyacrylamide gel electrophoresis. The PVDF membrane was blocked for 1 h at room temperature with 5% BSA before being incubated overnight at 4°C with primary antibodies including anti-BAX (Proteintech,1:500), anti-BCL2 (Proteintech, 1:500), anti-CC3 (Cell Signaling Technology, 1:500), and anti-β-Actin (Proteintech, 1:500). After washing with 1% TBST thrice, the membrane was incubated with secondary antibody (1:5000) at 37°C for 45 min. ECL reagents (Millipore, United States) were used to detect the immunoreactive bands, which were then visualized using image lab software (Bio-Rad, United States, Version 6.1.0) (https://www.bio-rad.com/zh-cn/product/image-lab-software). The average gray value was analyzed by ImageJ (National Institutes of Health, United States, Version 1.0) (https://imagej.net/downloads).

### 2.13 Measurement of reactive oxide species

#### 2.13.1 The measurement of reactive oxide species in heart tissues

The frozen sections preparation of heart tissue is described in the literature (Arcega et al., 2019). The sections were incubated with a 12 μM DHE (Dihydroethidium) fluorescent probe (BestBio, China) for more information, according to the kit’s instructions. A fluorescence microscope (Nikon, Japan) was then used to photograph the sections. ImageJ was used to assess the intensity of the fluorescence.

#### 2.13.2 The measurement of reactive oxide species in neonatal mouse cardiomyocytes

##### 2.13.2.1 The measurement of reactive oxide species using flow cytometry

NMCMs were incubated for 45 min without light with a 5 μM DHE fluorescent probe at 37°C. After washing with PBS, the cells were digested with 0.25% trypsin (Gibco, United States). Approximately 5 × 10^5^ cells were collected for flow cytometry (FCM). The cells in the blank group were not stained with DHE, and the other treatments were the same as those described above.

##### 2.13.2.2 The measurement of reactive oxide species using a specific fluorescent probe

NMCMs were incubated for 45 min without light with a 5 μM DHE fluorescent probe at 37°C. After washing with PBS, the plate was placed into a microplate reader with a 535 nm excitation and 610 nm emission wavelength.

### 2.14 Measurement of cell apoptosis rate

After being digested by trypsin, the collected NMCMs were washed with PBS and then suspended in 500 μl of diluted 1 × annexinV binding buffer. The suspension was incubated with 5 μl Annexin V-FITC at room temperature for 15–20 min and 5 μl propidium iodide (PI) for another 5 min. The mixed solution was measured by FCM immediately after the reaction was completed.

### 2.15 Elisa

The serum samples from mice receiving various treatments were centrifuged and diluted before use. Then, according to the instructions, ELISA kits (Elabscience, China) were used to analyze the serum inflammatory cytokine levels of TNF-α and IL-6. The standard curve of each inflammatory cytokine was used to calculate the level of inflammatory cytokines in the serum of mice.

### 2.16 Statistical analyses

GraphPad Prism (Insightful Science, United States, Version8.0.1) (https://www.graphpad-prism.cn/) was used to conduct statistical analyses and diagrams. All data were presented as the mean ± SD. Two-tailed t-tests were used for two-group comparisons, and one-way or two-way analysis of variance (ANOVA) was used for multigroup comparisons. Statistical significance was defined as *p* < 0.05.

## 3 Results

### 3.1 Bioactive components in Renshen Yangrong decoction

The TCMSP database was used to screen out 56 bioactive components of RSYRD, including 3-acetoxyatractylone, sitosterol naringenin, hepta-3, inermin, kaempferol, isorhamnetin, worenine, and others (Supplementary Table S1).

### 3.2 Potential targets of Renshen Yangrong decoction

**FIGURE 1:**
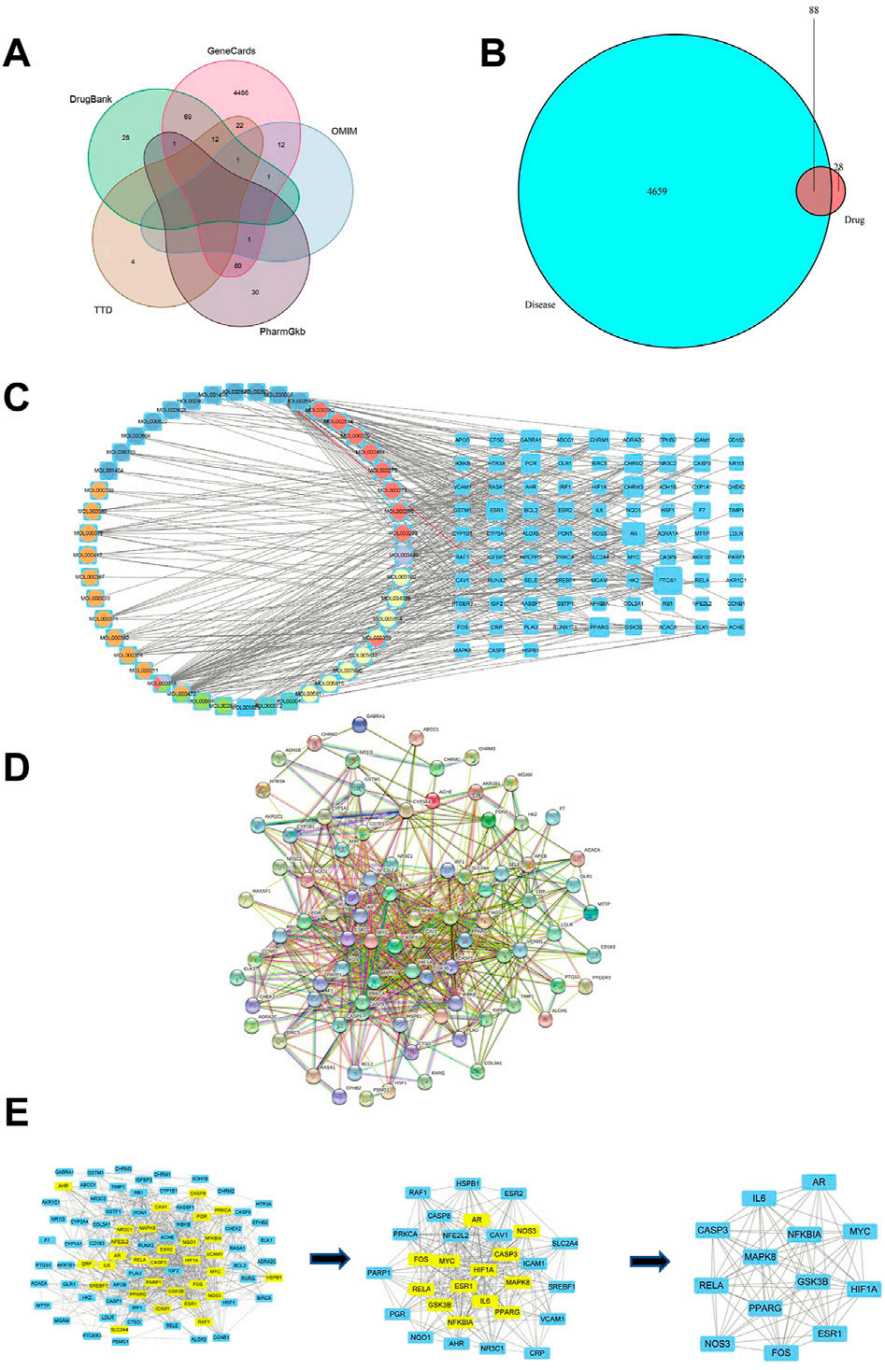
Network construction and PPI network core screening. **(A)** Venn diagram of the five databases surveyed. **(B)** The intersections between targets associated with RSYRD and MI. **(C)** Network of bioactive compounds—targets—disease. Yellow-orange: Hedysarum Multijugum Maxim.; blue: Coptidis Rhizoma; orange-red: Poria Cocos (Schw.) Wolf.; purple: Angelicae Sinensis Radix; green blue: Atractylodes Macrocephala Koidz.; green: Panax Ginseng C. A. Mey. **(D)** PPI network of intersections. **(E)** Screening of the PPI network core.

Venn diagram analysis was available to obtain the intersections between RSYRD and MI bioactive components. There were 116 potential targets identified from 56 bioactive components. A total of 4747 MI-related genes were identified using the GeneCards, OMIM, PharmGkb, TTD, and Drugbank databases (Figure 1A). Eighty-eight intersections were found (Figure 1B).

### 3.3 Regulatory network construction and hub targets

To identify hub proteins regulated by RSYRD in MI, the bioactive components of RSYRD were matched with the intersections above (Figure 1B) to build a regulatory network (Figure 1C). The protein-protein interaction network was constructed using the online String Database (Figure 1D). The importance of components and intersections was evaluated according to the degree of connectivity between them in the network. β-sitosterol, kaempferol, nobiletin, quercetin, (R)-canadine, palmatine, 7-O-methylisomucronulatol, calycosin, 3,9-di-O-methylnissolin, formononetin, isorhamnetin play a vital role in the regulatory network of RSYRD and were closely related to numerous intersections (Figure 1C). AR, PPARG, ESR1, CASP3, IL-6, MAPK8, MYC, HIF1A, RELA, NOS3, FOS, GSK3B, and NFKBIA were the hub targets. They played an essential role in the regulatory network, indicating that they might be involved in the development of MI (Figure 1E).

### 3.4 GO and KEGG enrichment analyses

**FIGURE 2:**
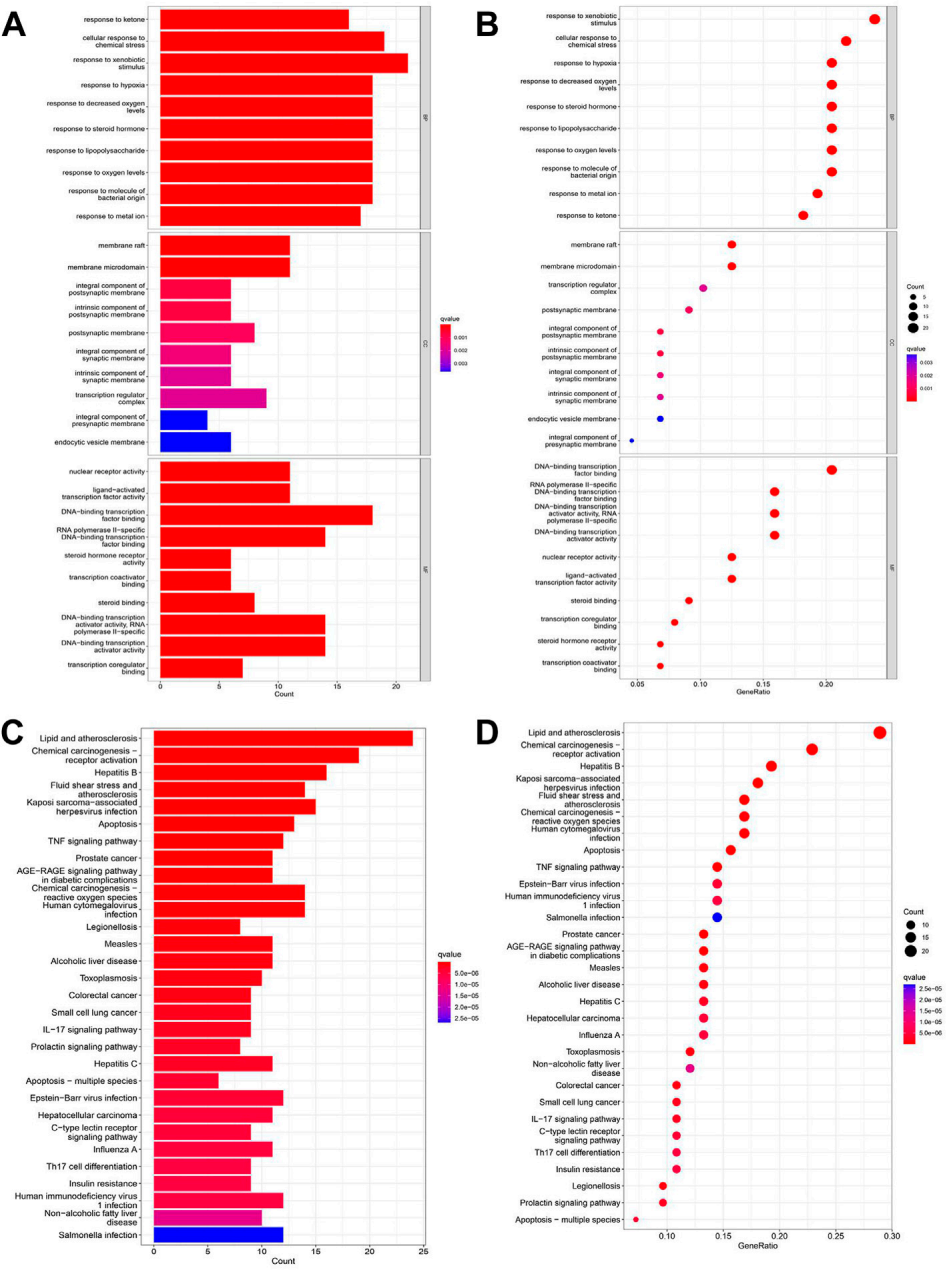
GO and KEGG enrichment analyses of intersections. **(A)** Barplot of GO annotation which shows the top 10 significantly enriched terms in molecular function (MF), biological process (BP), and cellular component (CC) respectively. **(B)** Bubble diagram of GO annotation which shows the top 10 significantly enriched terms in MF, BP, and CC respectively. **(C)** Barplot of KEGG enrichment analysis which shows the top 30 significantly enriched terms in the KEGG pathway. **(D)** Bubble diagram of KEGG enrichment analysis which shows the top 30 significantly enriched terms in the KEGG pathway. Gene ratio = count/set size.

We used GO and KEGG functional enrichment analyses to investigate the potential signaling pathways of these intersections. The screening criteria were as follows: the *p* value cutoff was 0.05, and the Q value cutoff was 0.05. Finally, We identified 1305 biological processes using GO functional enrichment analysis (Supplementary Table S2). We used GO functional enrichment analysis to annotate gene functions, including biological processes (BPs), cellular components (CCs), and molecular functions (MFs). BP primarily included the response to ketone, the response to hypoxia, the response to decreased oxygen levels, etc. The majority of these responses were associated with hypoxic injury in MI. CC mainly included membrane rafts, membrane microdomains, integral components of the postsynaptic membrane, and so on. MF mainly included nuclear receptor activity, ligand-activated transcription factor activity, DNA-binding transcription factor binding, and so on (Figures 2A,B).

One hundred twenty-seven signaling pathways were obtained *via* KEGG pathway enrichment analysis, including lipid and atherosclerosis, chemical carcinogenesis receptor activation, hepatitis B, fluid shear stress, atherosclerosis, kaposi sarcoma−associated with hepatitis, virus infection, apoptosis, and TNF signaling pathway (Supplementary Table S3; Figures 2C,D). The evidence suggests that RSYRD may play an essential role in cell apoptosis and the inflammatory response in MI.

In conclusion, the effects of RSYRD on MI were primarily associated with oxidative stress injury, inflammatory response, and apoptosis.

### 3.5 Three days after myocardial infraction, apoptosis and reactive oxide species increased in myocardial tissue

**FIGURE 3:**
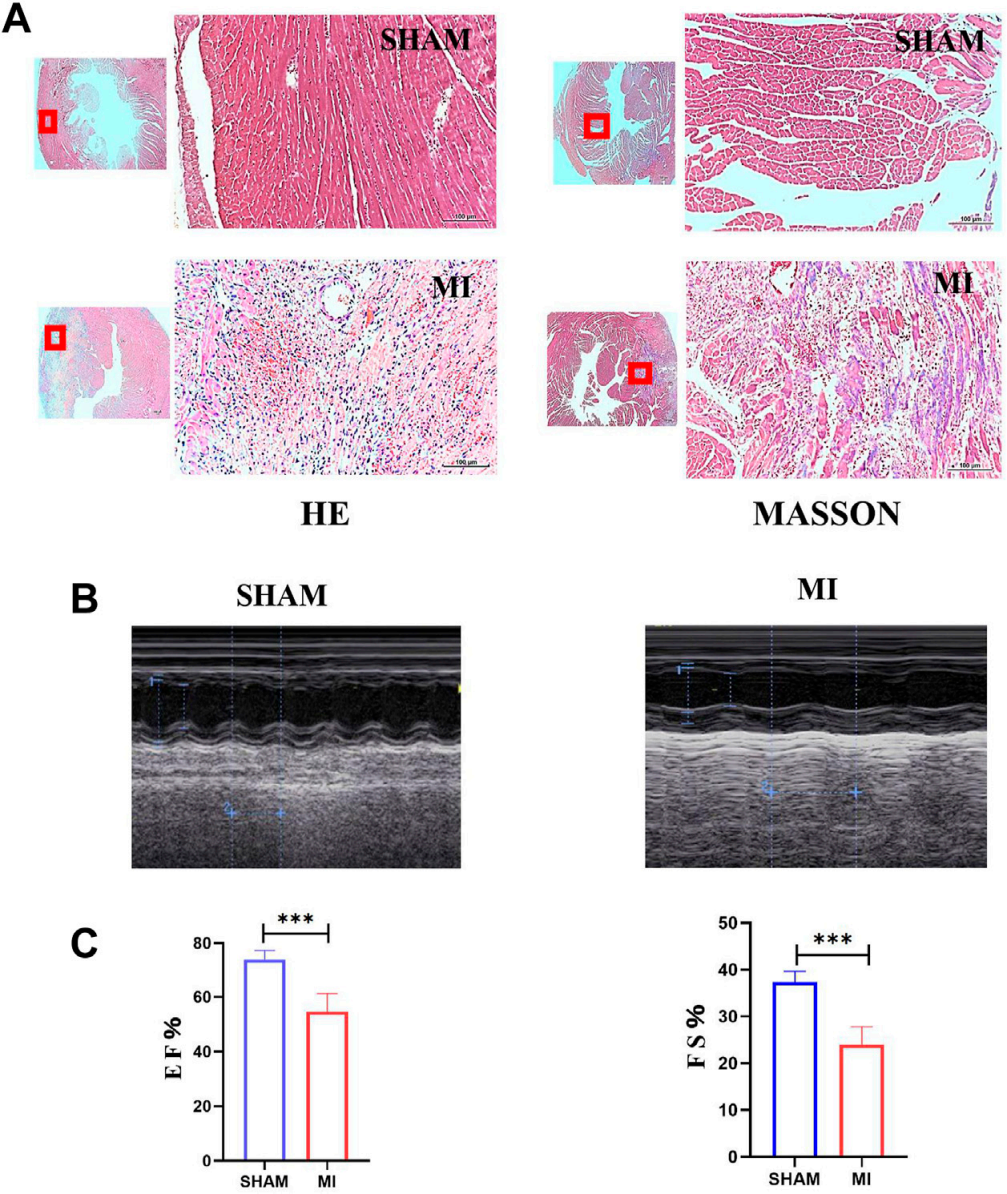
Establishment of the MI model of mice. **(A)** H&E and Masson staining of hearts of mice in SHAM and MI groups. **(B)** Ultrasonic detection of heart function in mice in the SHAM and MI groups. **(C)** FS and EF of mice in SHAM and MI groups (mean ± SD, *n* = 5). ***, *p* < 0.001; Scale bars: 100 μm.

**FIGURE 4:**
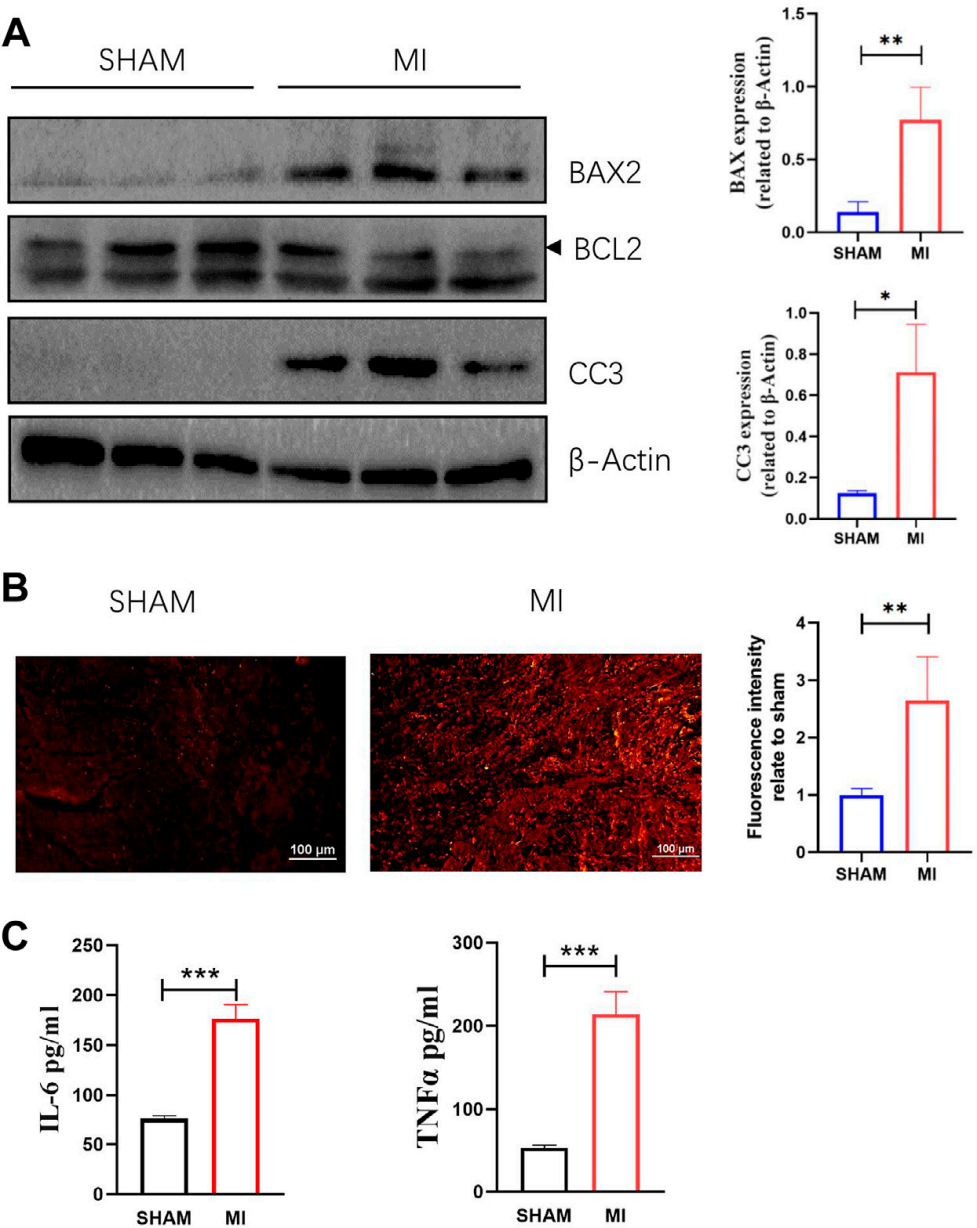
Apoptosis, ROS production in myocardial tissue, and levels of TNF-a and IL-6 in serum are increased 3 days after MI. **(A)** Western blotting shows the relative protein intensities of BAX2, BCL2, and CC3 (mean ± SD, *n* = 3). **(B)** ROS fluorescence intensity of frozen heart sections from mice in the SHAM and MI groups (mean ± SD, *n* = 5). **(C)** The levels of IL-6 and TNF-a in serum of mice in the SHAM and MI groups (mean ± SD, *n* = 5). *, *p* < 0.05; **, *p* < 0.01; ***, *p* < 0.001; Scale bars: 100 μm.

A mouse model of MI was established to investigate apoptosis and ROS production changes in myocardial tissue during the early stages of MI. The hearts of mice were harvested and stained 3 days after the operation. Masson and H&E staining results revealed that the myocardial tissue at the infarct site was loosely arranged and infiltrated by immune cells. However, the tissue in the SHAM group was unharmed (Figure 3A). The cardiac functions of mice were evaluated to confirm whether the LAD was successfully ligated. The ultrasound detection results revealed that EF and FS in the MI group were significantly lower than in the SHAM group (Figures 3B,C), indicating successful LAD ligation and impaired normal heart function after the operation. The results of WB revealed that the expression of BAX and CC3 was significantly increased in the MI group, while the expression of BCL2 was significantly decreased (Figure 4A). ROS production was significantly increased in the MI group, according to the DHE fluorescence results (Figure 4B). According to the evidence above, apoptosis and ROS production in myocardial tissue increased dramatically in mice 3 days post-MI. Furthermore, IL-6 and TNF-α levels in the serum of mice in the MI group were significantly elevated (Figure 4C).

### 3.6 Apoptosis and reactive oxide species production of neonatal mouse cardiomyocytes were significantly increased after hypoxic injury

**FIGURE 5:**
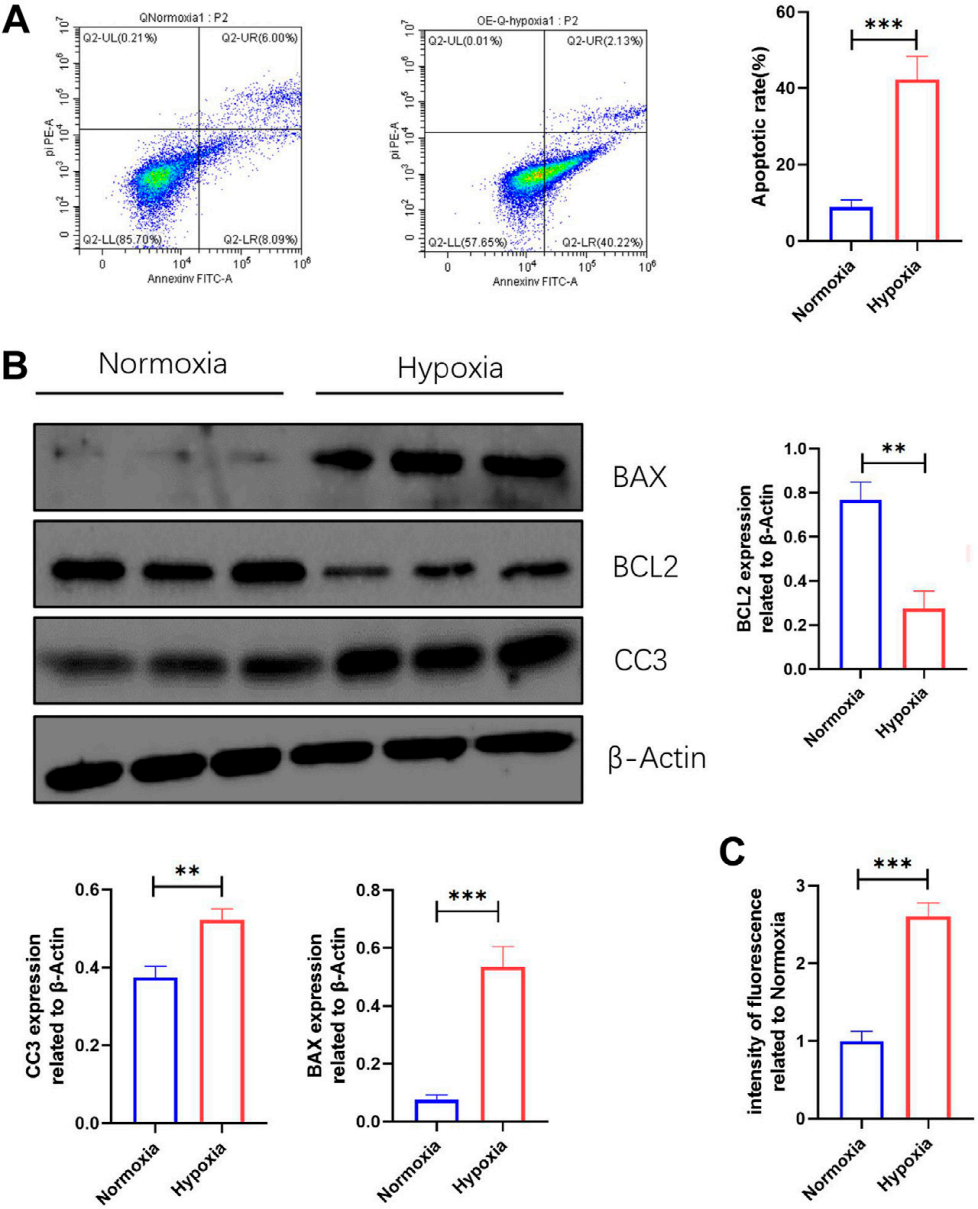
Apoptosis and ROS production are increased in NMCMs in response to hypoxia. **(A)** Apoptosis rate in Normoxia and Hypoxia groups (mean ± SD, *n* = 3). **(B)** Western blotting shows protein intensities of BAX2, BCL2, and CC3 in NMCMs (mean ± SD, *n* = 3). **(C)** ROS fluorescence intensity of NMCMs in Normoxia and Hypoxia groups measured by a microplate reader (mean ± SD, *n* = 8). **, *p* < 0.01; ***, *p* < 0.001.

The changes in ROS production and apoptosis of myocardial tissue *in vivo* were evident, but we do not know how they change *in vitro*. The FCM results revealed that the apoptosis rate of NMCMs induced by hypoxia was significantly increased (Figure 5A). WB results showed that BAX and CC3 expression were significantly increased, while BCL2 expression was significantly decreased in NMCMs in response to hypoxia (Figure 5B). The changes at the molecular and cellular levels indicated that apoptosis increased significantly in hypoxia-induced NMCMs. Microplate reader measurements, as before, revealed that in response to hypoxia, ROS production in NMCMs increased significantly (Figure 5C). All of these findings suggest that hypoxia could increase the apoptosis rate of NMCMs as well as the production of ROS.

### 3.7 Renshen Yangrong decoction improved cardiac function after myocardial infraction and alleviated cardiomyocyte apoptosis

**FIGURE 6:**
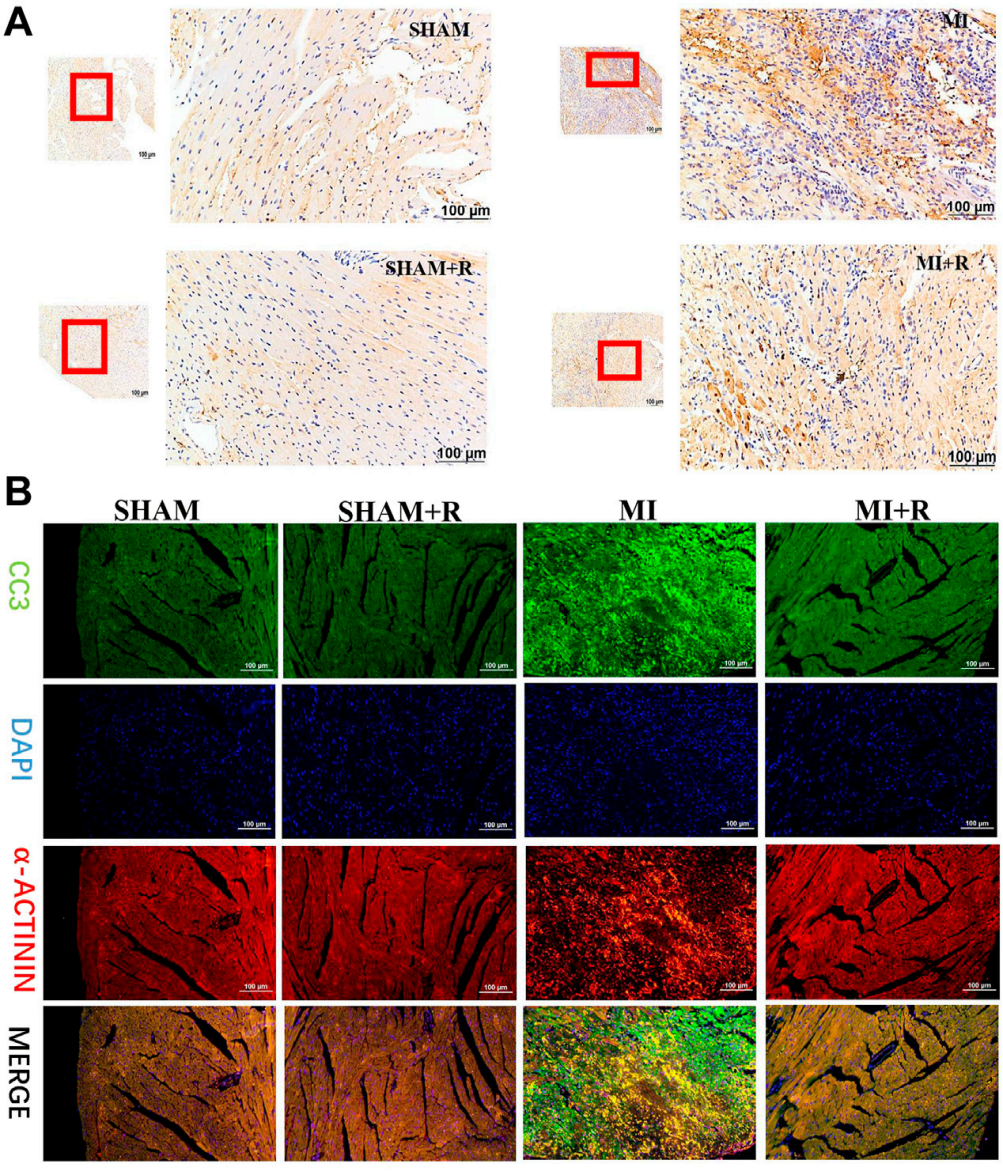
RSYRD can reduce the expression of CC3 in the myocardial tissue 3 days after MI. **(A)** IHC staining for CC3 in myocardial tissue of mice in four groups. **(B)** IF staining for CC3 in myocardial tissue of mice in four groups. Scale bars: 100 μm.

**FIGURE 7:**
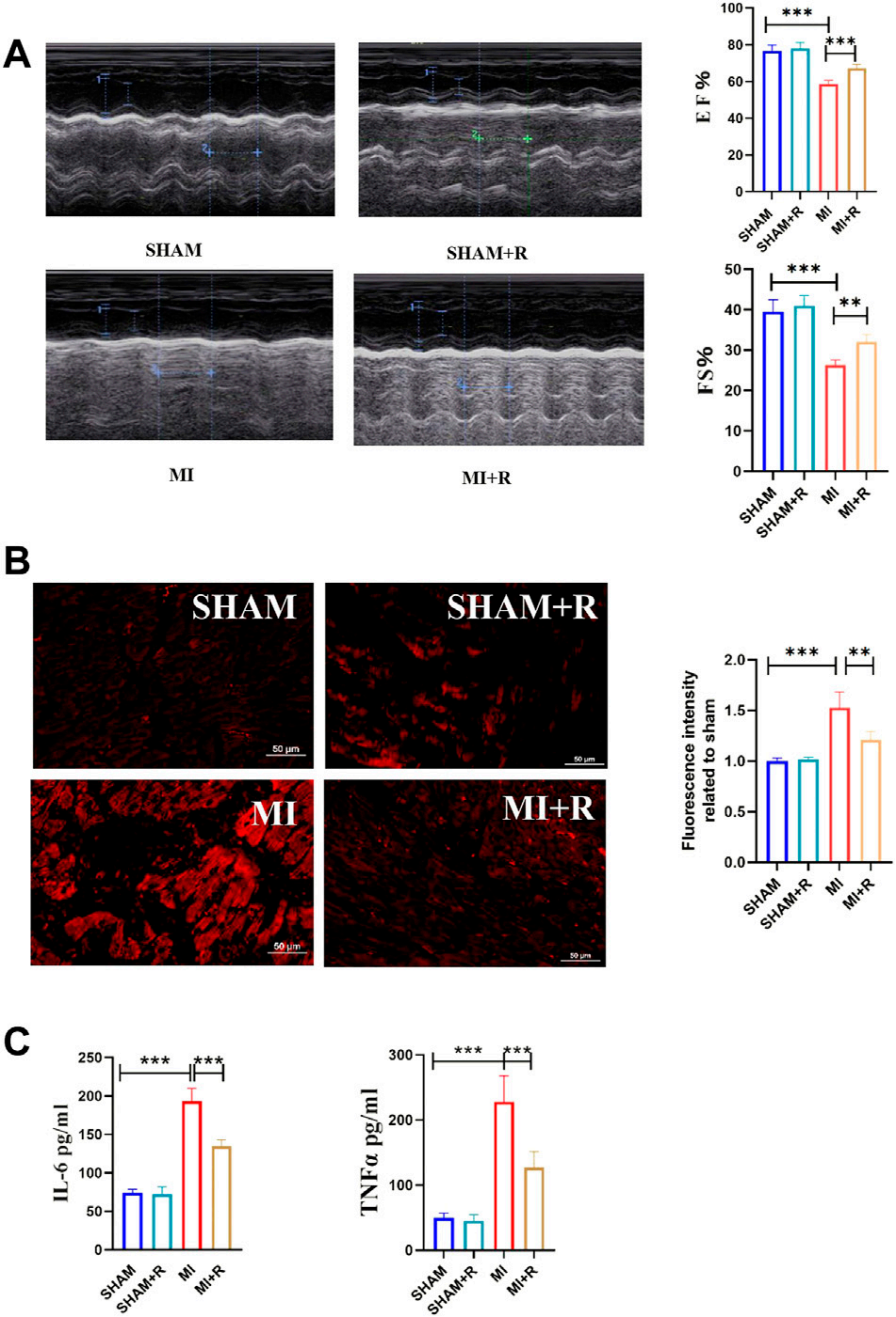
RSYRD can reduce the production of ROS, and inflammatory cytokine levels and improve heart function. **(A)** EF and FS of mice in four groups (mean ± SD, *n* = 5). **(B)** ROS fluorescence intensity of mice heart tissue in four groups (mean ± SD, n = 3). **(C)** Levels of IL-6 and TNF-a in serum of mice in four groups (mean ± SD, *n* = 5). **, *p* < 0.01; ***, *p* < 0.001; Scale bars: 50 μm.

Mice were treated with RSYRD to evaluate its effects on MI. IHC and IF staining results showed substantially lower CC3 expression in the MI + R group (Figures 6A,B). Compared to the MI group, the ultrasound detection results showed significantly higher EF and FS in the MI + R group (Figure 7A); and ROS staining results showed significantly lower ROS fluorescence intensity in the MI + R group (Figure 7B). Compared with the MI group, IL-6 and TNF-α levels were also reduced in the MI + R group (Figure 7C).

These findings suggest RSYRD can decrease myocardial apoptosis, reduce ROS production, and improve cardiac function after MI. Similarly, IL-6 and TNF-α decreased dramatically following RSYRD treatment, demonstrating that RSYRD had an anti-inflammatory effect.

**FIGURE 8:**
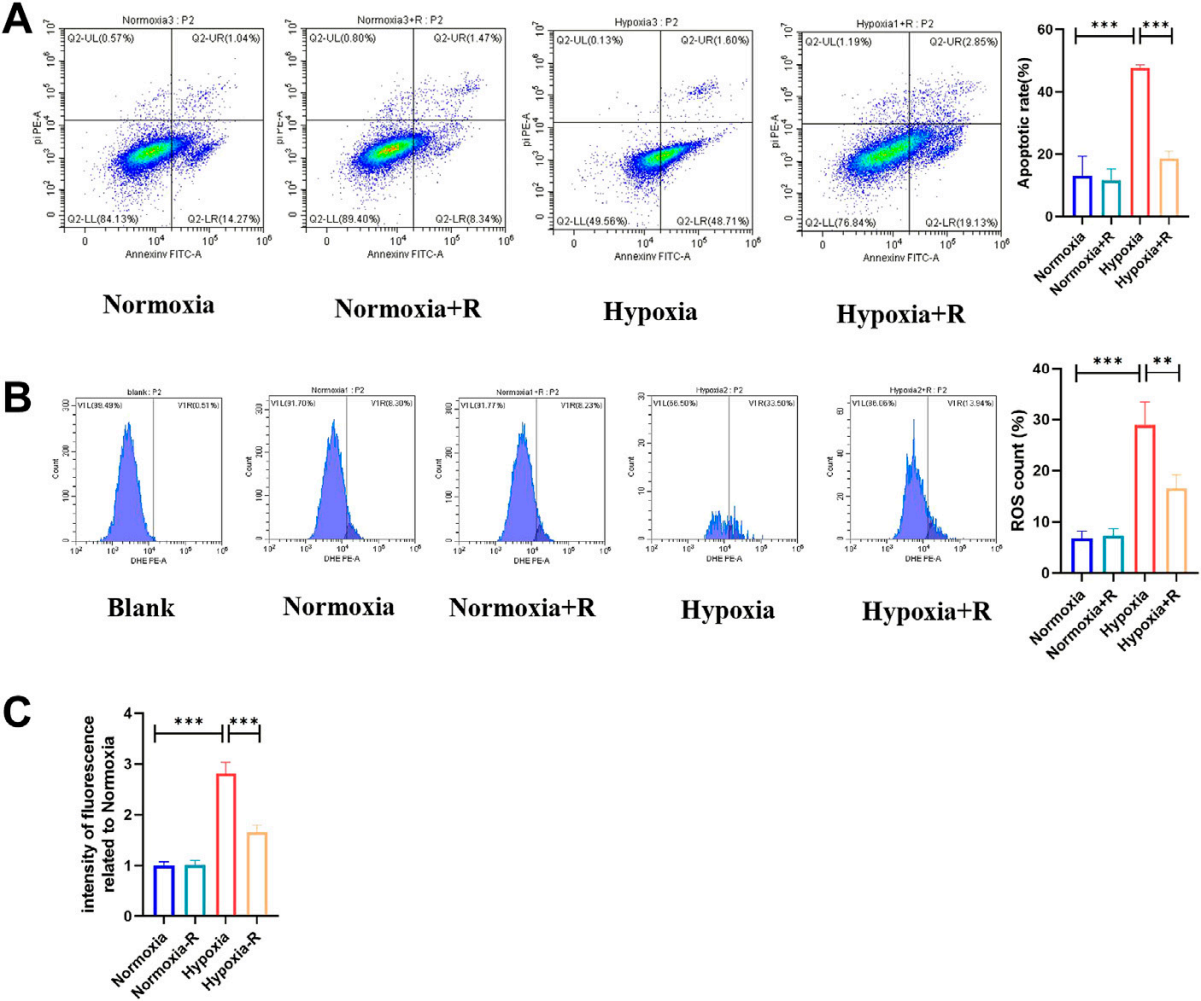
RSYRD can reduce the apoptosis rate and ROS production of NMCMs stimulated by hypoxia. **(A)** The apoptosis rate of NMCMs in four groups (mean ± SD, *n* = 3). **(B)** ROS production of NMCMs in four groups (mean ± SD, *n* = 3). **(C)** ROS fluorescence intensity of NMCMs in four groups measured by a microplate reader (mean ± SD, n = 8). **, *p* < 0.01; ***, *p* < 0.001.

*In vitro*, compared to the Hypoxia group, the cell apoptosis rate in the Hypoxia + R group was significantly reduced, as shown by the FCM results (Figure 8A). ROS was decreased considerably, as demonstrated by the microplate reader measurement results, indicating that RSYRD could reduce cell apoptosis and ROS production caused by hypoxia (Figure 8B).

### 3.8 Renshen Yangrong decoction reduced apoptosis by reducing the production of reactive oxide species in hypoxic environment

Multiple factors, including ROS, can induce apoptosis in hypoxia (Coimbra et al., 2017). The results of experiments *in vivo* and *in vitro* revealed that ROS concurrently increased with cell apoptosis and that RSYRD could alleviate this phenomenon. As a result, we hypothesized that RSYRD could reduce cell apoptosis by lowering ROS production.

**FIGURE 9:**
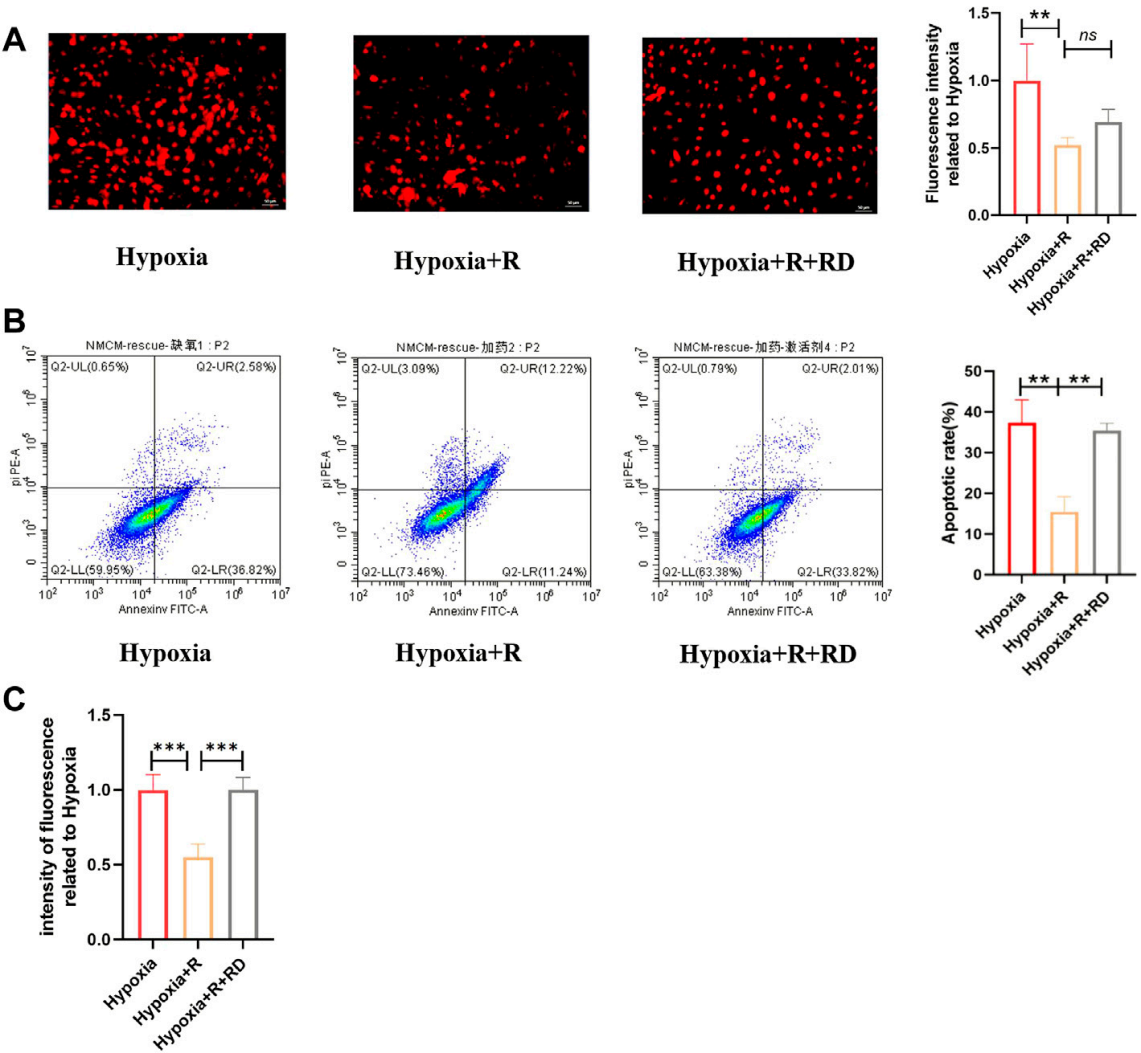
RSYRD can reduce ROS production to reduce the apoptosis rate of NMCMs. **(A)** ROS fluorescence intensity of NMCMs in three groups (mean ± SD, *n* = 3). **(B)** Apoptosis rates of NMCMs in three groups (mean ± SD, *n* = 3). **(C)** ROS fluorescence intensity of NMCMs in three groups detected by a microplate reader (mean ± SD, *n* = 8). **, *p* < 0.01; ***, *p* < 0.001; Scale bars: 50um.

According to the hypothesis, compared to the H + R group, the ROS production and apoptosis rate in the H + R + RD group increased, indicating that RSYRD plays a protective role in cells by inhibiting ROS production (Figure 9).

## 4 Discussion

RSYRD is essential in the treatment of cardiovascular diseases, but its effect on MI has not been studied. According to this study, RSYRD contained 56 bioactive components, including β-sitosterol, naringenin, quercetin, and others. β-sitosterol protects against myocardial ischemia/reperfusion injury (IRI) by targeting the PPAR/NF-κβ signaling pathway (Lin et al., 2020) and plays a protective role in inhibiting the inflammatory response and oxidative stress in both myocardial and renal IRI (Koc et al., 2021). Another study has found that β-sitosterol protects H9C2 cells and rat hearts from oxidative stress by increasing cellular glutathione activity (Wong et al., 2014). In mice, naringenin can increase SIRT1 expression, reduce oxidative stress injury to the myocardial tissue, lower cardiovascular risk, and prevent myocardial cell senescence (Testai et al., 2013; Da et al., 2017). Naringin protects against cardiac hypertrophy by activating ETS-PPARs (Zhang et al., 2018; Zhang et al., 2019). Naringin also reduces myocardial IRI *via* the CTGMP-PKGI pathway (Yu et al., 2019). Numerous studies have shown that quercetin can activate mitochondrial K^+^ channels while inhibiting TGF-/Smads or SIRT1/TMBIM6 signaling pathways, increasing cardiomyocyte tolerance to hypoxia and improving cardiac function (Testai et al., 2013; Chang et al., 2021; Wang et al., 2021). All of these findings suggest that RSYRD may be beneficial in MI.

According to the KEGG and GO enrichment results, the intersections were closely related to the oxidative stress and apoptosis pathways. We hypothesized that RSYRD might play a role in the development of MI by regulating these biological processes. We also found 13 hub genes, including CASPASE3, an enzyme that mediates cell apoptosis (Nagata, 2018; Eskandari et al., 2022). Increased ROS production caused apoptosis, implying that RSYRD might regulate apoptosis in myocardial tissue by regulating ROS levels in MI (Huang et al., 2022). This study’s findings revealed increased myocardial apoptosis and ROS in MI. However, after MI, RSYRD treatment reduced ROS and cell apoptosis, and activation of ROS reversed the apoptosis alleviated by RSYRD. Our speculation was confirmed by the evidence presented above.

In addition to oxidative stress and apoptosis signaling pathways, KEGG and GO analyses revealed that the immune-inflammatory pathway (TNF signaling pathway) played a crucial role after MI, as previously reported in many publications (Prabhu and Frangogiannis, 2016). The results of the molecular network analysis also revealed that IL-6 was a hub protein. As a result, TNF-α and IL-6 were chosen as inflammatory markers. TNF-α inhibition after MI can reduce inflammation and improve heart function (Somasuntharam et al., 2016). Similarly, IL-6 can regulate the immune response. Inhibiting IL-6 signal transduction after an acute MI can reduce the risk of cardiovascular events (Fernández, 2021; Ridker, 2021).

TNF-α and IL-6 levels in mice serum increased significantly after MI but decreased significantly after the treatment of RSYRD, indicating that RSYRD could have an anti-inflammatory effect. However, the specific mechanism of RSYRD’s anti-inflammatory effect has not been elaborated and needs to be studied in future experiments. Furthermore, it is worth investigating whether TNF-α and IL-6 play a role in myocardial tissue apoptosis and whether RSYRD can reduce apoptosis after MI by lowering inflammatory cytokine levels.

Additional potential pathways, such as the IL-7 signaling pathway and the MAPK 8 signaling pathway, were discovered through KEGG and GO enrichment analyses. These predicted important targets and signaling pathways need to be investigated further. As a result, additional therapeutic targets for RSYRD need to be studied.

## 5 Conclusion

There were 88 intersections between targets associated with RSYRD and MI; 13 hub targets, including AR, PPARG, ESR1, CASP3, IL-6, MAPK8, MYC, HIF1A, RELA, NOS3, FOS, GSK3B, and NFKBIA, were identified. According to the GO and KEGG functional enrichment analyses, these intersections were closely related to oxidative stress injury, inflammatory response, and apoptosis signaling pathways. Experiments *in vivo* revealed that 3 days after MI, myocardial tissue apoptosis, ROS production, TNF-α, and IL-6 levels increased significantly. Experiments *in vitro* demonstrated that stimulation of NMCMs with hypoxia significantly increased their apoptosis rate and ROS production. RSYRD treatment reduced apoptosis, ROS production, TNF-α, and IL-6 levels in myocardial tissue and improved cardiac function in mice with MI. Similarly, treating hypoxia-induced NMCMs with mouse serum containing RSYRD could reduce ROS production and apoptosis rate. At the mechanistic level, RSYRD could reduce hypoxia-induced apoptosis by inhibiting production of ROS and play a cytoprotective role.

## Data Availability

The original contributions presented in the study are included in the article/Supplementary Material, further inquiries can be directed to the corresponding author.
