# Supplementary material for: Network pharmacology-based and experimental identification of the effects of Renshen Yangrong decoction on myocardial infarction
Source: Front Pharmacol. 2022 Oct 25;13:1010036. doi: 10.3389/fphar.2022.1010036 (PMC9641366; doi:10.3389/fphar.2022.1010036)
Supplement: Supplementary file 2 [file Table1.DOCX]

**Supplementary Table1.** The bioactive components in RSYRD

| **Mol ID** | **Molecule Name** | **OB (%)** | **DL** |
| --- | --- | --- | --- |
| MOL000049 | 3beita-acetoxyatractylone | 54.07 | 0.22 |
| MOL000072 | 8beita-ethoxy atractylenolide Ⅲ | 35.95 | 0.21 |
| MOL000359 | sitosterol | 36.91 | 0.75 |
| MOL004328 | naringenin | 37.21 | 0.68 |
| MOL007930 | hesperidin | 13.33 | 0.67 |
| MOL005100 | 5,7-dihydroxy-2-(3-hydroxy-4-methoxyphenyl)  chroman-4-one | 35.52 | 0.67 |
| MOL005811 | Hepta-3 | 23.91 | 0.58 |
| MOL005812 | naringin | 6.92 | 0.78 |
| MOL005814 | tangeretin | 21.38 | 0.43 |
| MOL005815 | Citromitin | 86.9 | 0.51 |
| MOL000358 | beta-sitosterol | 36.91 | 0.75 |
| MOL000449 | Stigmasterol | 43.83 | 0.76 |
| MOL000273 | (2R)-2-[(3S,5R,10S,13R,14R,16R,17R)  -3,16-dihydroxy-4,4,10,13,14-pentamethyl  -2,3,5,6,12,15,16,17-octahydro-1H-cyclopenta[a]  phenanthren-17-yl]-6-methylhept-5-enoic acid | 37.59 | 0.68 |
| MOL000275 | trametenolic acid | 38.71 | 0.8 |
| MOL000279 | Cerevisterol | 37.96 | 0.77 |
| MOL000282 | ergosta-7,22E-dien-3beta-ol | 43.51 | 0.72 |
| MOL000283 | Ergosterol peroxide | 40.36 | 0.81 |
| MOL000296 | hederagenin | 36.91 | 0.75 |
| MOL002464 | 1-Monolinolein | 37.18 | 0.3 |
| MOL002514 | Sexangularetin | 62.86 | 0.3 |
| MOL001454 | berberine | 36.86 | 0.78 |
| MOL002894 | berberrubine | 35.74 | 0.73 |
| MOL002897 | epiberberine | 43.09 | 0.78 |
| MOL002903 | (R)-Canadine | 55.37 | 0.77 |
| MOL002904 | Berlambine | 36.68 | 0.82 |
| MOL002907 | Corchoroside A_qt | 104.95 | 0.78 |
| MOL000622 | Magnograndiolide | 63.71 | 0.19 |
| MOL000785 | palmatine | 64.6 | 0.65 |
| MOL000098 | quercetin | 46.43 | 0.28 |
| MOL001458 | coptisine | 30.67 | 0.86 |
| MOL002668 | Worenine | 45.83 | 0.87 |
| MOL000211 | Mairin | 55.38 | 0.78 |
| MOL000239 | Jaranol | 50.83 | 0.29 |
| MOL000033 | (3S,8S,9S,10R,13R,14S,17R)-10,13-dimethyl  -17-[(2R,5S)-5-propan-2-yloctan-2-yl]  -2,3,4,7,8,9,11,12,14,15,16,17-dodecahydro-1H-cyclopenta[a]phenanthren-3-ol | 36.23 | 0.78 |
| MOL000354 | isorhamnetin | 49.6 | 0.31 |
| MOL000371 | 3,9-di-O-methylnissolin | 53.74 | 0.48 |
| MOL000378 | 7-O-methylisomucronulatol | 74.69 | 0.3 |
| MOL000380 | (6aR,11aR)-9,10-dimethoxy-6a,11a-  dihydro-6H-benzofurano[3,2-c]chromen-3-ol | 64.26 | 0.42 |
| MOL000387 | Bifendate | 31.1 | 0.67 |
| MOL000392 | formononetin | 69.67 | 0.21 |
| MOL000417 | Calycosin | 47.75 | 0.24 |
| MOL000422 | kaempferol | 41.88 | 0.24 |
| MOL002879 | Diop | 43.59 | 0.39 |
| MOL003648 | Inermin | 65.83 | 0.54 |
| MOL005308 | Aposiopolamine | 66.65 | 0.22 |
| MOL005317 | Deoxyharringtonine | 39.27 | 0.81 |
| MOL005318 | Dianthramine | 40.45 | 0.2 |
| MOL005320 | arachidonate | 45.57 | 0.2 |
| MOL005321 | Frutinone A | 65.9 | 0.34 |
| MOL005344 | ginsenoside rh2 | 32.12 | 0.56 |
| MOL005348 | Ginsenoside-Rh4_qt | 31.11 | 0.78 |
| MOL005356 | Girinimbin | 61.22 | 0.31 |
| MOL005376 | Panaxadiol | 61.22 | 0.31 |
| MOL005384 | suchilactone | 57.52 | 0.56 |
| MOL005399 | alexandrin_qt | 36.91 | 0.75 |
| MOL000787 | Fumarine | 59.26 | 0.83 |
